# Human Platelet-Rich Plasma Facilitates Angiogenesis to Restore Impaired Uterine Environments with Asherman’s Syndrome for Embryo Implantation and Following Pregnancy in Mice

**DOI:** 10.3390/cells11091549

**Published:** 2022-05-05

**Authors:** Min Kyoung Kim, Jung Ah Yoon, Sook Young Yoon, Mira Park, Woo Sik Lee, Sang Woo Lyu, Haengseok Song

**Affiliations:** 1 Department of Obstetrics and Gynecology, CHA Fertility Center Gangnam, CHA University School of Medicine, 569 Nonhyun-ro, Gangnam-gu, Seoul 06125, Korea; flowmoco@chamc.co.kr (M.K.K.); jayoon@chamc.co.kr (J.A.Y.); syyoon11@cha.ac.kr (S.Y.Y.); wooslee@cha.ac.kr (W.S.L.); dung5038@naver.com (S.W.L.); 2 Department of Biomedical Science, CHA University, 335 Pangyo-ro, Bundang-gu, Seongnam 13488, Korea; mirapark@chauniv.ac.kr

**Keywords:** Asherman’s syndrome, platelet-rich plasma, infertility, uterine regeneration, angiogenesis

## Abstract

Asherman’s syndrome (AS) is caused by intrauterine adhesions and inactive endometrium from repeated curettage of the uterine endometrium. AS is a major cause of recurrent implantation failure and miscarriage and is very difficult to treat because of the poor recovery of endometrial basal cells. Platelet-rich plasma (PRP) has abundant growth factors that may induce angiogenesis and cell proliferation. Here, we demonstrate that human PRP (hPRP) significantly enhances angiogenesis to restore embryo implantation, leading to successful pregnancy in mice with AS. In mice with AS, hPRP treatment considerably reduced the expression of fibrosis markers and alleviated oligo/amenorrhea phenotypes. Mice with AS did not produce any pups, but the hPRP therapy restored their infertility. AS-induced abnormalities, such as aberrantly delayed embryo implantation and intrauterine growth retardation, were considerably eliminated by hPRP. Furthermore, hPRP significantly promoted not only the elevation of various angiogenic factors, but also the migration of endometrial stromal cells. It also increased the phosphorylation of STAT3, a critical mediator of wound healing, and the expression of tissue remodeling genes in a fibrotic uterus. PRP could be a promising therapeutic strategy to promote angiogenesis and reduce fibrosis in impaired uterine environments, leading to successful embryo implantation for better clinical outcomes in patients with AS.

## 1. Introduction

Asherman’s syndrome (AS) is characterized by amenorrhea caused by intrauterine adhesions and inactive endometrium from repeated curettage of the uterus after pregnancy or abortion [1]. AS is a major cause of recurrent implantation failure (RIF) and is very difficult to treat due to the poor recovery of endometrial basal cells. The common characteristics of AS are poor vascular development, poor gland growth, intrauterine adhesions, and altered expression of adhesion-related cytokines, resulting in a low embryo implantation rate [2]. Various treatments—such as hysteroscopy, estrogen supplementation, colony-stimulating factors, and stem cell therapy—have resulted only in minor improvements in endometrial thickness and clinical pregnancy outcomes [3,4,5]. Autologous bone-marrow-derived mesenchymal stem cells (MSCs) have been suggested as the potential source for endometrial regeneration [6,7]. However, owing to the invasiveness of collecting MSCs from the bone marrow, these MSCs are not quite easily available for many patients with AS. Nonetheless, MSCs can be obtained from the umbilical cord, amnion, and adipose tissue, but the availability remains controversial and they show limited fertility improvements in patients with AS [8]. Therefore, alternative therapeutic options that are easily accessible and less invasive are needed to improve the endometrial environment in patients with AS.

**Figure 1:**
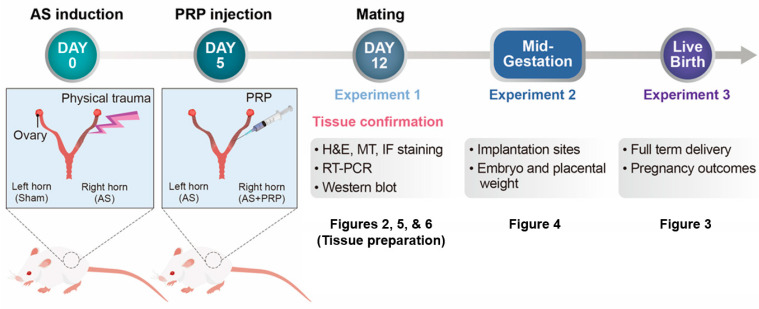
A schematic time-line to show the procedures of experiments performed in this study. Three independent experiments were performed in a mouse model of human Asherman’s syndrome (AS) to evaluate therapeutic effects of hPRP on the injured endometrium. Uterine tissues were prepared 12 days after AS was induced by physical insults (5 days after PRP treatment) for Figures 2, 5, and 6. Mice with AS were housed with fertile males to evaluate reproductive cycle in these mice and pregnant mice were separately maintained until live pups were delivered in Figure 3. Lastly, embryo implantation and post-implantation embryo development in mice with AS were observed on days 5 and 12 of pregnancy.

Platelet-rich plasma (PRP) is defined as serum containing more than 1,000,000 platelets per cubic microliter. PRP is rich in several growth factors that may induce angiogenesis and cell proliferation [9]. These factors include vascular endothelial growth factor (VEGF), platelet-derived growth factor, transforming growth factor, and epidermal growth factor [10]. These growth factors regulate various cell functions, including attachment, migration, proliferation, and extracellular matrix accumulation [11]. Thus, PRP therapy has been performed to promote tissue regeneration not only in many pathological conditions in various tissues—such as muscle, eye, and bone—but also in burns, trauma, and postoperative recovery [12,13,14,15]. Its most important benefit is that it is easy to obtain from the patient (blood collection by simple venipuncture) and ready to use within 30 min after centrifuge. Recently, PRP treatment provided better clinical results in AS patients undergoing in vitro fertilization (IVF) [16,17,18,19]. However, the molecular mechanisms of PRP-induced regeneration of damaged endometrium leading to better pregnancy rates are not yet fully understood. Thus, this study aims to elucidate the underlying mechanisms of PRP-induced endometrial regeneration in AS patients using a murine AS model (Figure 1) and human endometrial stromal cells. In this study, we demonstrate that PRP from patients with AS significantly facilitates uterine angiogenesis to restore impaired endometrium for successful embryo implantation and postimplantation fetal development in a murine model of AS.

## 2. Materials and Methods

### 2.1. Animals

All experiments were approved by the Institutional Animal Care and Use Committee of CHA University (IACUC 200070). Seven-week-old female ICR mice were purchased from Samtako (Seoul, Korea). Mice were housed in the Animal Care Facility of CHA University according to the institutional guidelines for laboratory animals under temperature-controlled conditions with a 12-h daily light/dark cycle and fed ad libitum.

### 2.2. PRP Preparation

Human PRP collection for experimental use was approved by the CHA University Gangnam CHA Hospital institutional review board (GCI NON2020-004). PRP sample was obtained from healthy female donors with history of RIF (who were scheduled for PRP treatment) and no diagnosed blood diseases. Blood was collected using citrate dextrose solution A as the anticoagulant, and PRP was prepared from the blood sample using a PRS Bio Kit (PTODIZEN, Inc., Seoul, Korea). A 150 μL sample from each donor was sent to the laboratory to confirm the platelet concentration. The mean platelet concentration in the original and the PRP samples was 266,000 cells/μL and 4,500,500 cells/μL, respectively.

### 2.3. PRP Therapy to Experimentally Induced Murine Model of AS

Seven-week-old ICR female mice were used to generate the murine model of AS that we previously described with slight modifications [20,21,22] (Figure 1). After administration of avertin (2,2,2-tribromoethanol, Acros Organics, NJ, USA) by intraperitoneal injection, a vertical incision was made in the abdominal wall, and the uterus was exposed. A small incision was made in each uterine horn at the utero-tubal junction and bilateral injury to the uterine horns was induced; a 27-gauge needle was inserted through the lumen, rotated, and withdrawn 10 times. PRP was delivered into one or both uterine horn(s) 5 days after AS induction depending on experimental purposes. Seven days after PRP injection, the uterine samples were collected for RT-PCR, Western blot, and/or histological analyses.

### 2.4. Analyses of Embryo Implantation and Pregnancy Outcomes

To evaluate the effects of human PRP on pregnancy outcomes in mice with AS, we housed female mice with AS together with fertile males for mating 7 days after PRP injection. A vaginal plug found on the next morning was considered day 1 of pregnancy. ISs on days 5 and 6 of pregnancy were visualized by intravenous injection (0.1 mL/mouse) of Chicago blue dye solution (1% in PBS, Sigma-Aldrich, St. Louis, MO, USA), and the number of ISs demarcated by distinct blue bands was recorded. The uteri without ISs were flushed with medium to recover unimplanted blastocysts. The number of ISs was counted, and the gross morphology and weight of fetal embryos and their placenta were examined on pregnancy day 12. For evaluating reproductive performance, mice with AS, with or without PRP treatment, were individually bred with fertile males and the numbers of pups per litter were counted.

### 2.5. RNA Extraction and Quantitative Real-Time RT-PCR (qRT-PCR)

Total RNA was extracted from each uterus using Trizol Reagent (Life Technologies, San Diego, CA, USA), according to the manufacturer’s protocols. First-strand cDNA was synthesized from 2 μg of total RNA using M-MLV reverse transcriptase and RNasin Ribonuclease Inhibitor (both Promega, Madison, WI, USA). Expression levels were measured using qRT-PCR with iQ SYBR Green Supermix (Bio-Rad, Hercules, CA, USA) on a Bio-Rad iCycler. To compare transcript levels between samples, a standard curve of cycle thresholds for several serial dilutions of a cDNA sample was established and used to calculate the relative abundance of each gene. Ribosomal protein L7 (rPL7) was used as a reference. All PCR reactions were performed in duplicate, and Supplementary Table S1 shows the primers used in the qRT-PCR assays.

### 2.6. Histological Staining

Uterine tissues were collected and fixed in 4% paraformaldehyde for histology. Fixed tissues were washed, dehydrated, and embedded in Paraplast (Merck KGaA, Darmstadt, Germany). Paraffin-embedded uterine tissues were sectioned and stained with hematoxylin and eosin (H&E) and Masson’s trichrome (MT). Uterine sections were treated with 0.01 M sodium citrate buffer (pH 6.0) for antigen retrieval. Sections were then incubated at 4 °C overnight with the following antibodies: anti-collagen type I (NB600-408, 1:200; Novus Biologicals, Charles, MO, USA), Ki-67 (ab166667, 1:200; Abcam, Cambridge, UK), anti-CD31 (553370, 1:200; BD Bioscience, Franklin Lakes, NJ, USA), anti-pSTAT3 (pSTAT3 (MA5-11189, 1:100, Thermo Fisher Scientific, Rockford, IL, USA), and Desmin (sc-23879, 1:50, Santa Cruz Biotechnology, CA, USA). On the following day, sections were washed with PBS and incubated with the appropriate secondary antibodies conjugated to fluorescein isothiocyanate (FITC, TRITC; 1:200; Jackson ImmunoResearch, West Grove, PA, USA). After three washes in PBS, sections were counterstained with 4′,6-diamidino-2-phenylindole (DAPI, Thermo Fisher, Schwerte, Germany) and propidium iodide (PI, Thermo Fisher, Schwerte, Germany) for nuclear staining. Images were obtained with an Axio Imager 2 microscope with ZEN 2012 software (Carl Zeiss, Jena, Germany).

### 2.7. Western Blot Analyses

Uterine tissues were lysed in 150 μL lysis buffer containing PRO-PREP Protein Extraction Solution (iNtRON, Seongnam, Korea) and 1× phosphatase inhibitor (Roche Applied Science, Indianapolis, IN, USA). Lysates were separated by 10% sodium dodecyl sulfate polyacrylamide gel electrophoresis (SDS-PAGE) (10 μg/lane), transferred onto polyvinylidene fluoride (PVDF) membranes (Bio-Rad), and blocked with 5% non-fat milk (Bio-Rad) in tris-buffered saline (Bio-Rad) containing 0.1% Tween 20 (TBST) (Sigma-Aldrich). Membranes were incubated overnight at 4 °C with appropriate antibodies (Supplementary Table S2). Goat-anti-rabbit, -mouse, or -rat IgG horseradish peroxidase (HRP)-conjugated secondary antibodies were diluted (1:3000) in TBST with 5% milk and used for 1 h at room temperature (RT, 25 °C). The signals were developed using the Clarity ECL Western blotting substrate kit and detected using a ChemiDoc XRS+ system with Image Lab software (version 4.0) (all Bio-Rad).

### 2.8. Scratch Wound-Healing Assay

A human endometrial stromal cell line (hESC, CRL-4003) was purchased from the American Type Culture Collection (ATCC; Manassas, VA, USA). Cells were seeded into 6-well plates at 2 × 10^5^ cells/well and cultured in Dulbecco’s modified Eagle’s medium: F12 without phenol red, supplemented with 10% fetal bovine serum and 1% antibiotic-antimycotic solution. Monolayers of confluent cells were scratched using a 100 μL pipette tip along a ruler without damaging the plastic and then washed twice with PBS to remove non-adherent cells. The medium was replaced with serum-free medium and the hESCs were cultured in six-well plates with inserts containing 2% human PRP or 2% serum [23]. We used an inverted microscope to monitor wound closure at 0, 6, 12, 18, and 24 h after the scratch and cell images were obtained at the same position. The wound areas in the cell migration assay were calculated using ImageJ (http://imageJ.nih.gov/ij/ accessed on 29 June 2020) [24].

### 2.9. Statistical Analysis

All data are shown as mean ± standard deviation. Statistical analyses were performed between two groups (sham vs. AS; AS vs. PRP) by using two-tailed Student’s *t*-tests by SPSS ver.26 (IBM, Armonk, NY, USA). In all cases, *p*-values < 0.05 were considered significant.

## 3. Results

### 3.1. PRP Significantly Reduces Fibrosis in the Uteri of Mice with AS

**Figure 2:**
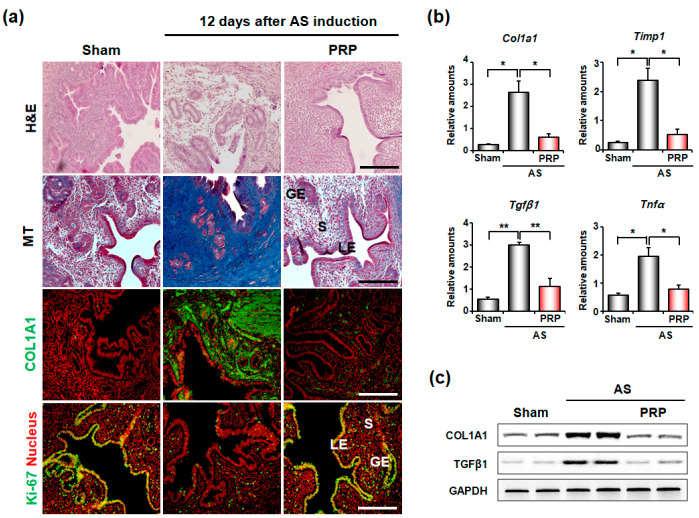
Intrauterine delivery of hPRP significantly reduces uterine fibrosis in mice with AS. (**a**) Histological analyses in AS uteri after intrauterine delivery of hPRP. Gross endometrial morphology and collagen deposition were evaluated by hematoxylin and eosin (H&E) staining and MT staining, respectively. Immunofluorescence staining of COL1A1 and KI-67 was performed to evaluate levels of collagen and proliferating cells, respectively. Green and yellow colors indicate COL1A1 and KI-67-positive cells, respectively; red indicates nuclei in uterine cells. LE, luminal epithelium; GE, glandular epithelium; S, stroma. Scale bar, 50 μm. (**b**,**c**) Real-time RT-PCR and Western blotting analyses for expression levels of fibrosis-related factors after hPRP treatment. * *p* < 0.05, ** *p* < 0.01.

Hematoxylin and eosin (H&E) and Masson’s trichrome (MT) staining clearly showed epithelial lining disruption and endometrial gland loss with severe fibrosis in mice with AS (Figure 2a). MT staining (blue color) and COL1A1 immunofluorescence staining (green color) demonstrated that collagen deposition significantly increased in the uteri of mice with AS, but was significantly reduced by PRP treatment. In Ki-67 staining (yellow color), epithelial cell amount remained very low in mice with AS, but it remarkably proliferated after PRP treatment. Furthermore, qRT-PCR (Figure 2b) and Western blotting (Figure 2c) analyses for fibrosis markers such as *Col1a1*, *Timp1*, *Tgfβ1*, and/or *Tnfα* quantitatively validated the significant reduction in uterine fibrosis in mice with AS treated with PRP. *Timp1* is known for suppressing collagenase activity [25], *Tgfβ1* plays significant role in the development of fibrosis by increasing extracellular matrix deposition [26] and *Tnfα* is an inflammatory cytokine that promotes cell differentiation in fibrotic tissues [27].

### 3.2. PRP Ameliorates Oligomenorrhea and Rescues Infertility of Mice with AS

**Figure 3:**
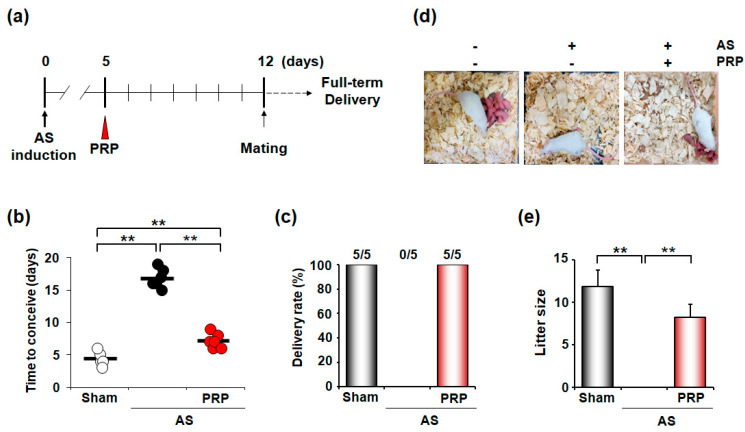
PRP treatment recovers irregular reproductive cycle and poor pregnancy outcome in mice with AS. (**a**) A schematic diagram to show the experimental procedures to examine the therapeutic effects of hPRP on the irregular reproductive cycle and infertility in mice with AS. (**b**–**e**) Therapeutic effects of hPRP on time to conceive (**b**), delivery rate (**c**), and litter size (**d**,**e**) in the pregnancy of mice with AS. The horizontal black lines represent median values. Numbers above the bars designate the number of mice that delivered live pup(s)/total number of examined mice. ** *p* < 0.01.

In evaluating the therapeutic effects of PRP on the clinical outcomes of mice with AS, we examined the time to conception, delivery rate, and litter size in mice with AS (Figure 3a). As expected, sham mice without AS took approximately 4–5 days to conceive, but the mice with AS took a considerably longer time, suggesting an oligomenorrhea-like phenotype in mice with AS. However, PRP-treated mice with AS showed a significantly shorter period to conceive (*p* < 0.001), although it did not reach the time of sham control (Figure 3b). All mice with AS failed to deliver (0/5), but all PRP-treated mice with AS (5/5) successfully delivered healthy live pups (*p* < 0.01) (Figure 3c). Accordingly, PRP-treated mice with AS produced live offspring, whereas mice with AS without PRP treatment did not (Figure 3d,e). Therefore, PRP provides beneficial effects to overcome infertility in mice with AS.

### 3.3. PRP Improves Poor Uterine Environment for Embryo Implantation Followed by Fetal Development in Mice with AS

**Figure 4:**
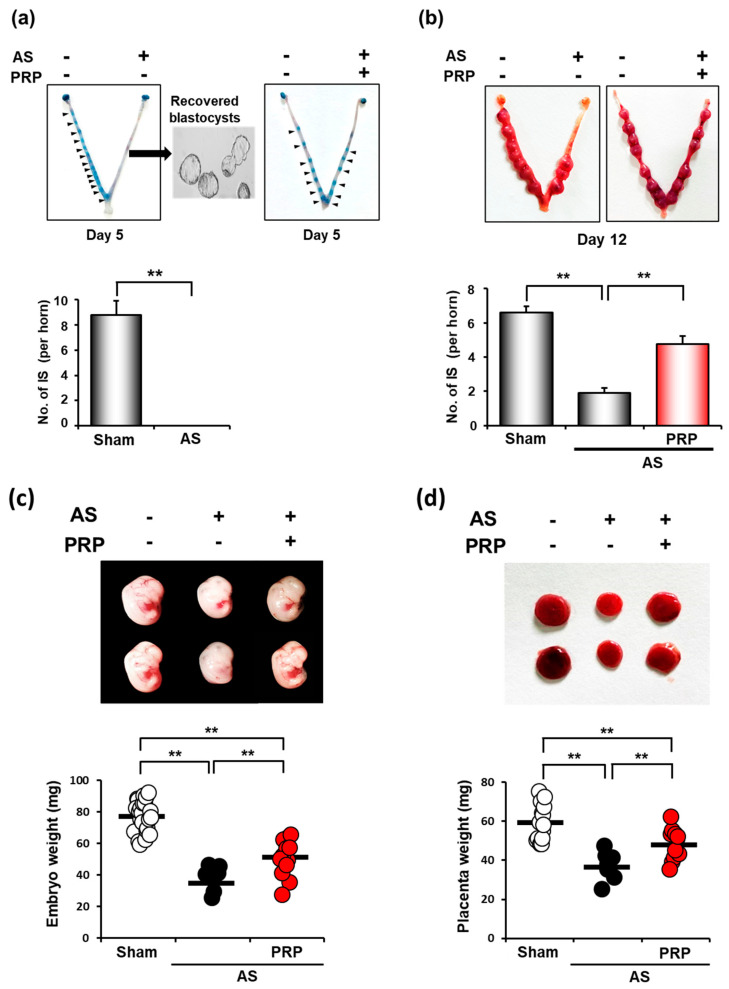
hPRP restores compromised uterine milieu for embryo implantation and subsequent pregnancy in mice with AS. (**a**) Failure of embryo implantation in the uterus of mice with AS in the morning of day 5 of pregnancy. Healthy unimplanted blastocysts were successfully retrieved from the uterus of mice with AS on day 5 of pregnancy. Note that hPRP therapy allows ‘on-time’ embryo implantation in mice with AS (blue bands along the right horn of the uterus with AS after hPRP treatment in the right box). (**b**) The representative images of the uterus and the number of implantations sites (ISs) in mice with AS after hPRP treatment on day 12 of pregnancy. Note that PRP treatment significantly increased the number of ISs in mice with AS on day 12 of pregnancy. (**c**,**d**) Representative images (upper images) and distribution graphs (bottom) of weights of embryos (**c**) and their placentas (**d**) that were harvested from ISs in mice with AS after hPRP treatment on day 12 of pregnancy. Note that hPRP treatment significantly increased the number of ISs and weights of embryos and placenta in mice with AS. The horizontal black lines represent the median values of weights of embryos (**c**) and their placentas (**d**). ** *p* < 0.01.

Embryo implantation is aberrantly delayed under suboptimal uterine conditions, such as AS, leading to intrauterine growth retardation (IUGR) and fetal loss [22,28]. Thus, we examined whether PRP treatment can recover unhealthy uterine conditions for ‘on-time’ embryo implantation in the next pregnancy. On pregnancy day 5, when the sign of embryo implantation is usually clearly observed (blue bands along the uterus), we did not find any implantation sites (ISs) in the uterus of mice with AS (Figure 4a). Healthy unimplanted blastocysts were retrieved from the uteri of mice with AS, suggesting that the uterine environment was unfavorable for embryo implantation in mice with AS. However, PRP treatment allowed embryo implantation in mice with such condition (the right uterine horn in the right uterine image).

While ISs were not observed at all on day 5 of pregnancy in mice with AS, we found ISs not only in sham control but also in the uterus with AS on day 12 of pregnancy, suggesting that embryo implantation was aberrantly delayed in mice with AS (Figure 4b). However, the number of ISs was significantly lower in mice with AS than in sham control, suggesting that such a delay in implantation may cause early pregnancy loss in the uterus with AS. Conversely, the number of ISs was significantly higher in the uterus with AS treated with PRP than in those without PRP treatment (*p* < 0.01), although it did not reach that of sham control. Embryos were collected on pregnancy day 12. In addition, the mean weights of embryos (*p* < 0.001) and their placenta (*p* < 0.001) from ISs (Figure 4c,d) were significantly increased in those treated with PRP, suggesting that PRP treatment considerably alleviates IUGR phenotypes in the uterus with AS.

### 3.4. PRP Promotes Angiogenesis and Cell Migration for Endometrial Regeneration and Remodeling in Mice with AS

**Figure 5:**
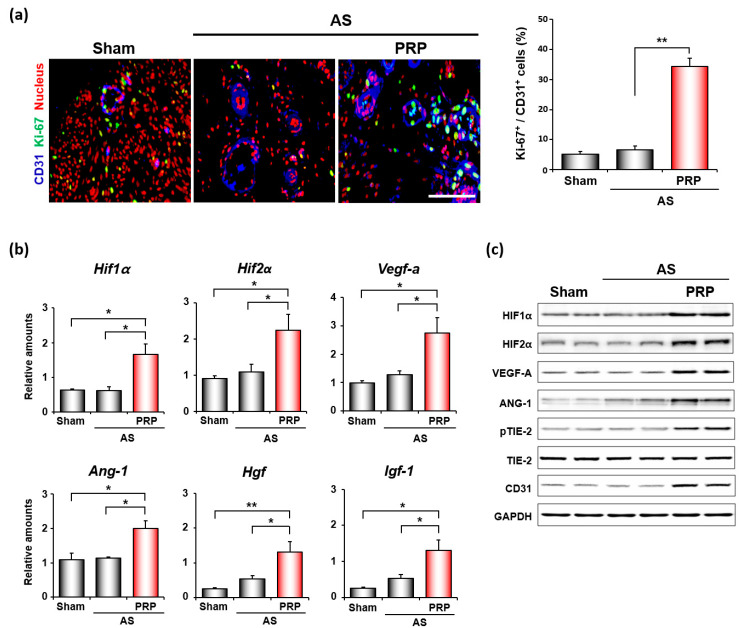
hPRP facilitates angiogenesis in compromised uterus in mice with AS. (**a**) Co-immunofluorescence staining of CD31 and KI-67 in uteri 7 days after intrauterine hPRP administration. Blue, green, and red colors indicate CD31, KI-67, and nuclei, respectively. Yellow nuclei indicate proliferating endothelial cells. The graph depicts the percentage of Ki-67 positive cells/CD31 positive cells counted. Scale bar, 50 μm. (**b**,**c**) Quantitative analyses of realtime RT-PCR (**b**) and Western blotting (**c**) of factors important for angiogenesis in uteri with AS after hPRP treatment. * *p* < 0.05, ** *p* < 0.01.

We next examined the underlying molecular mechanism(s) on how hPRP promotes uterine regeneration and alleviates infertile phenotypes for successful embryo implantation and pregnancy in mice with AS. Considering that PRP contains various growth factors to promote angiogenesis, we first performed co-immunofluorescence staining for CD31, which is an endothelial cell marker, and KI-67 for angiogenesis. As shown in Figure 5a, hPRP significantly increased the proliferation of endothelial cells in the uterus with AS. Consistent with increased uterine angiogenesis, hPRP therapy considerably increased the mRNA expression levels of proangiogenic factors such as *Hif1α*, *Hif2α*, *Vegf-a*, *Ang-1*, *Hgf*, and *Igf-1* (Figure 5b). *Hgf* is produced by stromal cells, and stimulates epithelial cell proliferation, motility, morphogenesis, and angiogenesis in various organs via tyrosine phosphorylation of its receptor, c-Met [29]. *Igf-1* is a growth factor responsible for cellular migration, proliferation, differentiation, and angiogenesis [30]. Furthermore, Western blotting analyses for proangiogenic factors—including ANG-1-dependent TIE2 phosphorylation, which is required for angiogenesis [31]—reinforced the angiogenic effects of hPRP at the protein levels in the uterus with AS (Figure 5c).

**Figure 6:**
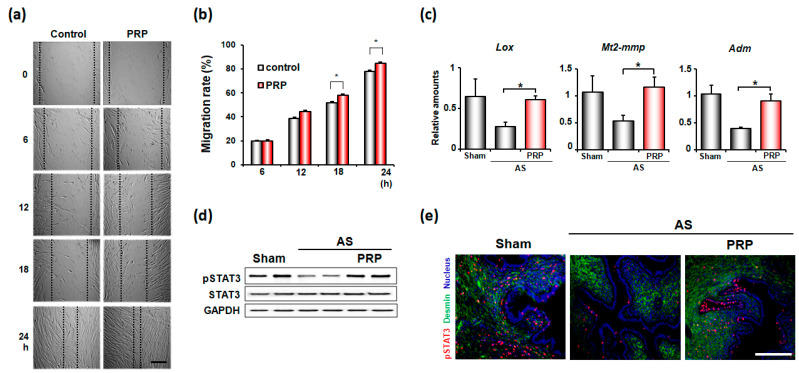
PRP promotes cell migration for endometrial remodeling in mice with AS. (**a**) Representative microscopic images of wound-healing assays with human endometrial stromal cells with or without hPRP for 24 h. Scale bar, 100 μm (**b**) The graphs for migration rates of endometrial stromal cells in (**a**). * *p* < 0.05. (**c**) Realtime-RT PCR results of tissue remodeling factors in the uterus with AS after hPRP treatment. (**d**,**e**) Western blotting (**d**) and immunofluorescence staining (**e**) of phosphorylated STAT3 (pSTAT3) in the uterus with AS after hPRP treatment. Red and blue colors indicate pSTAT3 and nucleus, respectively. Scale bar, 50 μm.

The effects of hPRP on endometrial cell migration in vitro were examined by scratch wound healing assay (Figure 6a). While hPRP did not show any effects at 6 h after treatment, it significantly increased endometrial cell migration afterwards (Figure 6b). Thus, PRP promotes the migration of endometrial stromal cells to injured uterine areas, leading to uterine regeneration in pathologic conditions, such as AS. In fact, hPRP treatment significantly increased Mt2-mmp, Lox, and Adm, which are known to be involved in tissue remodeling (Figure 6c). Furthermore, hPRP significantly increased the phosphorylation of STAT3, which is a critical transcription factor for tissue remodeling and regeneration, in both stroma and epithelial compartments in the uterus with AS (Figure 6d,e).

## 4. Discussion

PRP has gained growing attention for its therapeutic benefits in various tissue types—including bone, cartilage, tendon, and muscle—particularly in the context of traumatic injury [32]. We previously suggested hPRP’s beneficial effects in improving poor reproductive outcomes in a mouse model of human AS that reflects the clinical phenotypes of AS [33]. PRP treatment was recently applied to patients suffering from infertility with poor endometrial conditions, such as thin endometrium and AS [34,35,36]. Several clinical studies recently suggested that PRP has a therapeutic potential on the pregnancy outcomes of patients with infertility who underwent IVF. In patients with thin endometrium, PRP therapy significantly increased the endometrial thickness as well as pregnancy outcomes, including live birth rates [37,38,39]. Although these studies have relatively small sample sizes, they seem to clearly present that PRP significantly improves the uterine milieu of patients with infertility mainly caused by poor endometrium. Thus, understanding the underlying molecular mechanism of the positive effects of PRP on patients with thin endometrium and/or AS is crucial. In addition, PRP synergistically improves the therapeutic effects of menstrual blood-derived stromal cells on infertility in a rat model with intrauterine adhesion, possibly via the Hippo signaling pathway [37]. However, the mechanism on how PRP improves the suboptimal uterine environment in these patients remains undetermined. In the present study, we clearly demonstrated that hPRP promotes uterine angiogenesis, thereby restoring thin and or injured endometrium for successful embryo implantation and pregnancy in mice. 

Several main factors of PRP were known to have positive effects on uterine endometrium for fertility recovery. Platelet-derived growth factor has mitogenic effects in endometrial cells and induces wound repair by stimulating the chemotactic migration of endometrial stromal cells [40]. Epidermal growth factor triggers proliferation of endometrial cells and endometrial re-epithelialization [41]. IGF causes endometrial proliferation through the AKT/mTOR pathway and endometrial cell decidualization [42]. HGF enhances the endometrial regeneration during the menstrual cycle and induces endometrial epithelial cell proliferation [43]. We previously provided evidence that embryo implantation is aberrantly delayed in suboptimal uterine conditions, including AS [22,28,44]. The unsynchronized interactions between the blastocyst and unhealthy uterus, such as in AS, often lead to IUGR and/or pregnancy loss in both mice and humans [22,28,45]. Patients with AS have a relatively higher risk of developing IUGR during pregnancy [46,47]. In fact, the number of fetuses during pregnancy in mice with AS gradually decreased, indicating possible delivery failure (Figure 4). Thus, improper embryo implantation may result in infertility in patients with AS [47]. Providing PRP treatment before pregnancy in mice with AS reconditioned the compromised circumstance, allowing the blastocyst for on-time implantation (Figure 4a), suggesting that autologous PRP therapy is beneficial for patients suffering from RIF and recurrent spontaneous abortion, as well as AS. According to pilot studies using autologous PRP, PRP can be a therapeutic option for patients with poor endometrium [18,19,48,49,50].

Angiogenesis is a critical event for the endometrium, which undergoes repeated wound healing after menstruation [51,52]. In many pathological conditions, including AS, angiogenesis is crucial for increasing microvasculature and delivering sufficient oxygen and nutrients for tissue regeneration. In addition, extensive angiogenesis is required to provide uterine receptivity for successful embryo implantation [53]. Precedent endometrial injuries promoted endometrial angiogenesis to increase the potential of embryo implantation in mice [54]. We also previously demonstrated that HIF1α-dependent angiogenesis is a prerequisite for successful pregnancy following timely embryo implantation [22]. Defective angiogenesis may cause poor decidualization, possibly leading to recurrent pregnancy loss [55]. We showed that PRP treatment remarkably elevated angiogenesis by increasing the expression of several proangiogenic factors, such as *Hif1α* and *HIf2α*, in the uteri of mice with AS (Figure 5). Among the angiogenesis markers, HIF1α and HIF2α both induce VEGF, which regulates angiogenic switch, vascular permeability, and endothelial cell migration and proliferation [39]. In addition to angiogenic effects, PRP can effectively induce endometrial cell proliferation (Figure 2) and migration (Figure 6). Our results are supported by a previous study, which also showed that PRP improved the proliferation, migration, and/or adhesion of endometrial cells and bone marrow MSCs for endometrial regeneration [23,56]. Additionally, the transplantation of autologous menstrual blood-derived stromal cells leads to the migration of stromal cells to the adhesion sites, thereby increasing endometrial thickness in patients with AS [57]. Therefore, PRP facilitates the same and/or similar signaling pathways that MSCs trigger to help recover an injured endometrium.

We also found that PRP significantly increased the expression of *Mt2*-*mmp*, *Lox*, and *Adm*, which are involved in tissue remodeling in the uteri of mice with AS (Figure 6c). MT2-MMP induces the degradation of the extracellular matrix of basement membranes, cleaving epithelial E-cadherin [58,59]. LOX also debilitates the extracellular matrix of basement membranes [60]. Thus, MT2-MMP and LOX, which are both transcription targets of HIF [61,62], possibly prepare the endometrium in accepting embryo implantation. Lastly, ADM is an angiogenic, anti-inflammatory, and vasodilatory protein that supports endometrial implantation of the embryo by boosting the connexin 43-dependent gap junction in the stroma [63]. HIF2α-knockout mice with failed implantation exhibited decreased expression of VEGF, ADM, and LOX in the uterus [64]. The transcription factor STAT3 is also crucial because it has differential roles in uterine epithelial and stromal cells to coordinate uterine receptivity and embryo implantation [65]. Transcriptome analyses of a human endometrium showed that LIF-STAT3 signaling is compromised in the endometrium of patients with RIF [66]. In addition, a regenerating uterine epithelium showed heightened STAT3 phosphorylation and cell proliferation, which was suppressed in the uteri of *STAT3*-knockout mice [67]. Thus, heightened STAT3 phosphorylation in both the stromal and epithelial compartments of a regenerating uterus after PRP treatment (Figure 6d, 6e) suggests that PRP contains critical factors that activate major regenerating signals, such as STAT3 phosphorylation, for uterine regeneration in patients with AS.

## 5. Conclusions

Autologous PRP could be a beneficial and promising therapeutic option for preventing complications associated with a hostile endometrial environment, such as IUGR and miscarriage, for better clinical outcomes in patients with infertility who suffer from RIF, especially those with AS. Further human randomized controlled trials involving a large number of study participants are needed to confirm this conclusion.

## Data Availability

The datasets used and/or analyzed during the current study are available from the corresponding author on reasonable request.

## Supplementary Materials

The following supporting information can be downloaded at: https://www.mdpi.com/article/10.3390/cells11091549/s1, Table S1: Primers for RT-PCR and real-time RT-PCR, Table S2: List of antibodies used for Western blotting.
