# Supplementary material for: Human Platelet-Rich Plasma Facilitates Angiogenesis to Restore Impaired Uterine Environments with Asherman’s Syndrome for Embryo Implantation and Following Pregnancy in Mice
Source: Cells. 2022 May 5;11(9):1549. doi: 10.3390/cells11091549 (PMC9101537; doi:10.3390/cells11091549)
Supplement: Supplementary file 1 [file cells-11-01549-s001.zip › cells-1694671-supplementary.pdf]

Supplementary Table S1. Primers for RT-PCR and real-time RT-PCR

	Forward (5'→3')	Reverse (5'→3')	Amplicon (bp)
Col1a1	CTGGCGGTTCAGGTCCAAT	TTCCAGGCAATCCACGAGC	141
Timp1	GGGTTCCCCAGAAATCAACGAG	ACAGAGGCTTTCCATGACTGGGGTG	139
Tgfβ1	GTGAAACGGAAGCGCATCGAAG	CATAGTAGTCCGCTTCGGGCTCC	193
Tnfα	CTGAACTTCGGGGTGATCGG	GGCTTGTCACTCGAATTTTGAGA	122
Hif1α	ACAAGTCACCACAGGACAG	AGGGAGAAAATCAAGTCG	168
Hif2α	AATGACAGCTGACAAGGAGAAAAA	GAGTGAAGTCAAAGATGCTGTGTC	257
Vegf-a	GCAGGCTGCTGTAACGATGA	GCATGATCTGCATGGTGATGTT	105
Ang-1	GGGACAGCAGGCAAACAGA	TGTCGTTATCAGCATCCTTCGT	110
Hgf	CTGACCCAAACATCCGAGTTG	TTCCCATTGCCACGATAACAA	125
Igf-1	TGCTTCCGGAGCTGTGATCT	CGGGCTGCTTTTGTAGGCT	125
Lox	TTACACTAACAACGGCCGTGAAGA	CTAGACCACGGTCCCACTGAAGA	120
Mt2-mmp	GAGAGATGTTTGTGTTCAAGGG	TGTGTCAATGCGGTCATAGGG	260
Adm	GAGCGAAGCCCACATTCGT	GAAGCGGCATCCATTGCT	76
Rpl7	TCAATGGAGTAAGCCCAAAG	CAAGAGACCGAGCAATCAAG	246

Supplementary Table S2. List of antibodies used for Western blotting

Antibody	Company	Cat No.	Dilution
anti-collagen type I	Novus Biologicals	NB600-408	1:1000
anti-TGFβ1	Santa Cruz Biotechnology	sc-130348	1:1000
anti-hypoxia inducible factor (HIF)1α	Novus Biologicals	NB100-479	1:500
anti-HIF2α	Novus Biologicals	NB100-122	1:100
anti-VEGF	Abcam	ab46154	1:1000
anti-Ang1	R&D systems	MAB8220	1:1000
anti-pTIE-2	R&D systems	AF3909	1:1000
anti-TIE-2	R&D systems	AF762	1:1000
anti-CD31	BD Biosciences	553370	1:500
anti-pSTAT3	Cell signaling	9145	1:500
anti-STAT3	Thermo Fisher Scientific	MA5-15712	1:500
glyceradehyde-3-phosphate dehydrogenase (GAPDH)	Cell Signaling	2118	1:3000
